# Evolutionary and Functional Characterization of the Allene Oxide Cyclase (AOC) Gene Family Reveals the Joint Contribution of *GhAOC4/6* to Cotton Resistance Against *Verticillium dahliae*

**DOI:** 10.3390/plants15192914

**Published:** 2026-09-23

**Authors:** Jie He, Zhengwei Shao, Xinpei Han, Fengli Zhao, Yuan Wang, Xinjie Zhang, Yuanyuan Liu, Lu Yang, Zhanshuai Li, Jun Peng

**Affiliations:** 1 National Nanfan Research Institute (Sanya), Chinese Academy of Agricultural Sciences, Sanya 572025, China; 2 Institute of Cotton Research, Chinese Academy of Agricultural Sciences, Anyang 455000, China; 3 Sanya Institute of Nanjing Agricultural University, Sanya 572024, China; 4 Hainan State Farms Academy of Sciences Group Co., Ltd., Sanya 572025, China; 5 College of Agriculture, Tarim University, Alar 843300, China; 6 China National Rice Research Institute, Hangzhou 311401, China

**Keywords:** *Gossypium hirsutum*, allene oxide cyclase, *Verticillium dahliae*, jasmonic acid

## Abstract

AOC is a key enzyme in jasmonic acid (JA) biosynthesis and plays important roles in plant development and stress responses. However, the evolutionary characteristics and functional diversification of the *AOC* gene family in cotton remain poorly understood. In this study, *AOC* genes were identified and systematically characterized in four representative cotton species. Phylogenetic analysis revealed that cotton AOCs were mainly distributed in Clade II and Clade IV. Structural and promoter analyses indicated that cotton AOCs possess conserved Allene_ox_cyc domains but exhibit diversification in gene structures and regulatory elements. Synteny analysis revealed that tandem duplication and WGD/segmental duplication were the major driving forces underlying *AOC* gene family expansion in diploid and tetraploid cotton species, respectively. Expression analysis of *GhAOC* genes showed that *GhAOC6* and its homologous gene *GhAOC4* exhibited strong responses to *Verticillium dahliae* infection. Co-silencing of *GhAOC4/6* significantly reduced cotton resistance to *Verticillium* wilt. Overall, this study provides new insights into the evolutionary conservation and functional diversification of the *AOC* gene family in cotton and identifies the highly homologous *GhAOC4/6* pair as a candidate component associated with cotton resistance to *Verticillium* wilt.

## 1. Introduction

Plants are constantly exposed to various biotic and abiotic stresses in complex natural environments, among which infections by pathogenic microorganisms pose a serious threat to crop productivity. As a major economic crop, *Gossypium hirsutum* is severely affected by *Verticillium* wilt, a devastating disease caused by *V. dahliae*. This disease causes substantial yield losses and deterioration of fiber quality [1]. Therefore, elucidating the molecular mechanisms underlying cotton resistance to *Verticillium* wilt is essential for developing effective disease management strategies.

Plants orchestrate diverse hormonal responses through complex signaling networks, thereby activating defense mechanisms [2]. JA is a lipid derived phytohormone that plays critical roles in plant defense and development [3,4,5]. JA is biosynthesized from α-linolenic acid. α-Linolenic acid is first converted to 13(S)-hydroperoxy-(9Z,11E,15Z)-octadecatrienoic acid (13-HPOT) by lipoxygenase (LOX) [6]. 13-HPOT is then converted by allene oxide synthase (AOS) into an unstable allene oxide intermediate. This intermediate is subsequently cyclized by AOC to form 12-OPDA [7,8]. 12-OPDA is transferred to peroxisomes, where OPDA reductase 3 (OPR3) reduces it to 3-oxo-2-(2′(Z)-pentenyl)-cyclopentane-1-octanoic acid (OPC-8:0). OCP-8:0 is then converted to (+)-7-iso-JA through three β-oxidation steps. Subsequently, (+)-7-iso-JA is isomerized to (−)-JA and released into the cytosol by an unknown mechanism [9]. JA can be conjugated with isoleucine by JA-Ile synthase [10] to form JA-Ile [11]. Alternatively, JA can be methylated by JA carboxyl methyltransferase (JMT) to produce MeJA [12]. JA and JA-Ile can further regulate the expression of downstream transcription factors such as NAC, ERF, bZIP, and WRKY, thereby modulating the transcription of functional genes and enabling plants to cope with biotic and abiotic stresses [13,14,15].

AOC is a critical key enzyme in the JA biosynthesis pathway, and its expression is closely associated with JA accumulation. Therefore, *AOC* plays a central role in plant resistance to various biotic stresses. *ScAOC1* expression was significantly up-regulated in both susceptible (ROC22) and resistant (Yachen05179) sugarcane cultivars following infection with *Sporisorium scitamineum* [16]. However, the resistant cultivar exhibited higher transcript accumulation and longer duration of upregulation, suggesting that sustained high expression of *AOC* contributed to enhanced resistance to *Sporisorium scitamineum*. In tomato, JA biosynthesis genes, including *AOC*, were up-regulated by increased *SlMYC2* expression, thereby reducing susceptibility to *Botrytis cinerea* [17]. Overexpression of *MtAOC2* in alfalfa significantly increased JA content and enhanced resistance to *Rhizoctonia solani*, whereas *MtAOC2* mutants showed the opposite phenotype, further confirming that *AOC* positively regulated defense against this pathogen [18]. In rice, loss of function of *OsAOC* resulted in extremely low JA levels and increased susceptibility to *Magnaporthe oryzae* [19]; conversely, overexpression of *AOC* significantly enhanced resistance to the brown planthopper, indicating that *AOC* mediated JA synthesis was crucial for resistance to both fungal and insect pests [20]. Similarly, overexpression of *GmAOC3* in soybean significantly increased JA and JA-Ile contents, thereby enhancing insect resistance [21]. In tobacco, the highly conserved chloroplast protein cpSRP54 interacted with AOC and translocated it to the thylakoid membrane. This process promotes JA biosynthesis and ultimately enhances virus resistance [22]. Thus, *AOC* is broadly involved in plant defense against diverse biotic stresses, including pathogenic fungi, viruses, and insect pests, through the regulation of JA biosynthesis. Recent studies have further expanded the biological significance of AOC, highlighting its roles in pathogen susceptibility, insect resistance, and developmental regulation [23]. These findings indicate that *AOC* contributes to the coordination of plant defense and growth processes. In addition, *AOC* also plays important roles in plant growth and development [24,25] and responses to abiotic stresses such as osmotic stress [26], drought, salinity [27,28], low temperature [29,30] and wounding [31,32], highlighting its functional diversity and significance. In cotton, *GhAOC1* contributes to copper stress tolerance [33], and *GhAOC2* is required for male fertility under high temperature conditions [25]. More recently, *GmAOC4* was identified as a candidate gene associated with lint percentage in an introgression population [34]. However, a comprehensive characterization of the *AOC* gene family in cotton is still lacking. Such a systematic analysis would provide a basis for understanding the conservation and diversification of *AOC* genes in cotton and for identifying *AOC* members potentially involved in stress responses.

In this study, the *AOC* gene family was systematically identified and characterized in four representative *Gossypium* species. The chromosomal distribution, gene structure, conserved motifs, phylogenetic relationships, and synteny analyses were performed to investigate the evolutionary characteristics of cotton *AOC* genes. Together with the expression analyses, these analyses provided a systematic overview of the *GhAOC* family and a basis for selecting *GhAOC4* and *GhAOC6* for subsequent functional characterization. Using VIGS, simultaneous suppression of *GhAOC4/6* was found to compromise cotton resistance to *V. dahliae*.

## 2. Materials and Methods

### 2.1. Plant Materials and V. dahliae Treatments

The resistant cultivar ZZM2 of *Gossypium hirsutum* was cultivated in a greenhouse at the National Nanfan Research Institute of CAAS at 28 °C, 65% relative humidity, with a 16 h light/8 h dark photoperiod. The inoculation method for *V. dahliae* was adapted from previous studies, as detailed below [35]. The *V. dahliae* strain Vd991, preserved in our laboratory, was inoculated onto potato dextrose agar medium and cultured at 25 °C for 5 days. The activated Vd991 was then inoculated into Czapek–Dox liquid medium and cultured at 25 °C with shaking at 180 rpm for 7 days. A spore suspension of *V. dahliae* (1.0 × 10^7^ spores/mL) was prepared and inoculated with 10 mL onto the roots of each plant at the two leaves stage, following the procedure described by Ma et al. [36]. *Nicotiana benthamiana* seedlings were sown in nutrient soil and grown under the same conditions as the cotton plants.

### 2.2. Identification of the AOC Gene Family Members

Genomic data of *Gossypium hirsutum* and other species were obtained from the CottonOmics database (http://cotton.zju.edu.cn/index.htm, accessed on 5 February 2026), the National Center for Biotechnology Information (NCBI) database (https://www.ncbi.nlm.nih.gov/, accessed on 5 February 2026), and Integrated Medicinal Plantomics (IMP) database (https://www.bic.ac.cn/IMP/, accessed on 5 February 2026). *Arabidopsis thaliana* AOC (AT3G25760, AT3G25770, AT3G25780, AT1G13280) protein sequences were used as queries for BLASTP searches against the *Gossypium hirsutum* protein database, with an E-value cutoff of ≤1 × 10^−5^, to identify homologous sequences. The Hidden Markov Model (HMM) profile of the allene oxide cyclase domain (PF06351) was downloaded from the Pfam database (http://pfam.xfam.org/, accessed on 5 February 2026), and the *Gossypium hirsutum* protein database was screened for sequences containing this domain using TBtools (version 2.454) [37], with an E-value cutoff of ≤1 × 10^−5^. The candidate sequences obtained from BLASTP and HMM searches were combined, and redundant sequences were removed. All candidate proteins were subsequently examined using the NCBI Conserved Domain Database (CDD) to confirm the presence and completeness of the Allene_ox_cyc domain. Only proteins containing a complete Allene_ox_cyc domain were retained as AOC family members for subsequent analyses. Additional plant genomes were also screened during AOC family identification; however, species without identifiable AOC homologs were not included in subsequent analyses.

### 2.3. Multiple Sequence Alignment and Phylogenetic Analysis

AOC family protein sequences from 26 representative plant species were selected for phylogenetic analysis. These species were chosen to represent diverse plants lineages, including algae, bryophytes, pteridophytes, gymnosperms, monocots and dicots. Representative species from different taxonomic families were included whenever possible to capture the evolutionary diversity of AOC genes. Species lacking identifiable AOC homologs were excluded from the phylogenetic analysis. The selected AOC protein sequences were aligned using MAFFT with default parameters, and the resulting alignments were trimmed using trimAI with default parameters. A maximum likelihood phylogenetic tree was constructed using IQ-TREE with the Q.PFAM + G4 amino acid substitution model selected according to the Bayesian Information Criterion (BIC) and 5000 bootstrap replicates [38]. The resulting tree was rooted using the midpoint rooting method and visualized and annotated using the online tool iTOL (version 7.5) (https://itol.embl.de/, accessed on 15 February 2026).

### 2.4. Collinearity Analysis of AOC Genes in Ten Dicot Species

The R package syntenet (version 1.10.2) was used to infer synteny networks from whole genome protein sequences of ten dicotyledonous plants, including *Arabidopsis thaliana*, *Glycine max*, *Arachis hypogaea*, *Brassica napus*, *Nicotiana tabacum*, *Capsicum annuum*, *Gossypium raimondii*, *Gossypium arboreum*, *Gossypium hirsutum* and *Gossypium barbadense* [39]. The synteny results were visualized using NGenomeSyn (version 1.41) [40]. Intraspecific collinearity analysis and visualization were performed using TBtools (version 2.454). Phylogenetic relationships among species were inferred using the NCBI Common Taxonomy Tree tool. Duplication types of AOC genes, including tandem, proximal, dispersed, and WGD/segmental duplication, were identified using TBtools (version 2.454) with its default parameters. For Ka/Ks analysis, all AOC genes within four Gossypium species were paired in a pairwise manner, and Ka and Ks values were calculated using TBtools (version 2.454). Gene pairs with Ka = 0 and Ks = 0 were recorded as showing zero substitutions and were not assigned a numerical Ka/Ks ratio because the ratio is undefined. The duplication classification and Ka/Ks calculation results are provided in Tables S5 and S6, respectively. Chromosomal localization visualization was performed using TBtools (version 2.454).

### 2.5. Bioinformatics Analysis of AOC Genes in Cotton

Sequences of *AOC* gene family members were extracted from the cotton genome and annotation files using TBtools (version 2.454). Conserved motifs were analyzed using the MEME online tool (https://meme-suite.org/meme/, accessed on 8 March 2026), and conserved domains were identified with NCBI CDD. Gene structure and chromosomal localization were analyzed using TBtools (version 2.454). The 1500 bp region upstream of the start codon of each *AOC* gene was defined as the promoter region. *Cis*-acting regulatory elements were predicted by PlantCARE (https://bioinformatics.psb.ugent.be/webtools/plantcare/html/ accessed on 10 March 2026). The results were visualized using TBtools (version 2.454). Putative transcription factor binding sites were predicted using PlantTFDB (https://planttfdb.gao-lab.org/ accessed on 12 March 2026) with a stringent threshold (*p* ≤ 1 × 10^−6^), based on a set of high quality, non-redundant binding motifs from 156 species with whole genome sequences.

### 2.6. Analysis of GhAOC Expression Patterns

Publicly available RNA-seq data of *GhAOC* genes across different tissues and under various stress conditions were obtained from the CottonMD database (https://yanglab.hzau.edu.cn/CottonMD/ accessed on 15 March 2026) and a previous study [41]. Expression heatmaps were generated using the ImageGP tool (version 2.0) (https://www.bic.ac.cn/ImageGP/ accessed on 15 March 2026).

### 2.7. RNA Isolation and qPCR Analysis

Total RNA was extracted from plant tissue samples using the FastPure Universal Plant Total RNA Extraction Kit (Vazyme, Nanjing, China). RNA was reverse transcribed into cDNA using the Evo M-MLV Reverse Transcriptase Kit (Accurate Biotechnology, Changsha, China), and qPCR was performed using the SYBR Green Premix Pro Taq HS Fluorescent Quantitative PCR Kit (Accurate Biotechnology, Changsha, China). *GhUBQ7* was used as the internal reference gene, and the relative expression levels of target genes were calculated using the 2^−ΔΔCt^ method. Primer sequences are listed in Table S13.

### 2.8. Subcellular Localization

For subcellular localization analysis, the coding sequences of GhAOCs were fused to the N-terminus of eGFP, generating GhAOCs-eGFP fusion constructs. The empty vector expressing eGFP alone was used as the control. The recombinant plasmids were transformed into *Agrobacterium tumefaciens* GV3101, which was then infiltrated into *N. benthamiana* leaves for transient expression according to established protocols [42]. AtPIP2A-mCherry was used as a plasma membrane marker [43] and was co-infiltrated with the GhAOCs-eGFP constructs. Plasmolysis was induced by treating leaves with 0.8 M mannitol [44]. Fluorescence signals from eGFP, mCherry, and chloroplast autofluorescence were detected using laser scanning confocal microscopy (Leica Microsystems GmbH, Wetzlar, Germany).

### 2.9. Functional Analysis of GhAOC4/6 by VIGS

For VIGS mediated silencing of *GhAOC4/6*, a single fragment shared by *GhAOC6* and *GhAOC4* was selected based on their high sequence similarity. Sequence alignment of the *GhAOC* family members showed that the selected fragment was highly conserved between *GhAOC6* and *GhAOC4* but was distinct from the corresponding regions of the other *GhAOC* members (Figure S7). Therefore, this common *GhAOC4/6* fragment was cloned into the pTRV2 vector to generate the *GhAOC4/6* VIGS construct. Because *GhAOC4* and *GhAOC6* share an identical sequence within this fragment, the same VIGS construct simultaneously targeted both genes. Gene silencing in cotton was performed as previously described [45]. To evaluate the specificity of VIGS mediated *GhAOC4/6* silencing, transcript levels of all six *GhAOC* members identified in *G. hirsutum* were examined by RT-qPCR in control and *GhAOC4/6* silenced plants. The expression levels of the defense related genes *GhHIN1*, *GhHSR203* and *GhRBOHD* were analyzed in control and *GhAOC4/6* silenced plants before *V. dahliae* inoculation. The JA responsive genes *GhMYC2* and *GhPR4* were also analyzed under the same conditions.

H_2_O_2_ in cotton leaves was detected by DAB staining at 24 h after inoculation with *V. dahliae* [46]. Briefly, leaves were vacuum infiltrated with 1 mg/mL DAB solution for 30 min and incubated overnight at room temperature. After staining, chlorophyll was removed by incubation in 95% ethanol at 100 °C for 10 min, and the leaves were subsequently stored in 20% glycerol for observation using a stereomicroscope. Representative images were collected from each treatment. Three independent biological replicates were performed, with two plants analyzed per treatment in each replicate.

Lignin staining was examined using phloroglucinol as previously reported [47]. Briefly, stem sections were stained with 10% phloroglucinol solution for 2 min, followed by brief treatment with concentrated sulfuric acid. Representative images were obtained using a microscope, and lignin deposition was compared according to the relative intensity of phloroglucinol staining among treatments. Three independent biological replicates were performed, with two plants analyzed per treatment in each replicate.

Endogenous 12-OPDA, JA, and JA-Ile in cotton stems were quantified 15 days after *V. dahliae* inoculation using ultra performance liquid chromatography coupled with tandem mass spectrometry (UPLC-MS/MS; QSight LX50 coupled with QSight 420, PerkinElmer, Waltham, MA, USA). Briefly, approximately 0.3 g of frozen stem tissues were ground in liquid nitrogen and extracted with precooled acetonitrile containing isotope labeled internal standards. After ultrasonic extraction and centrifugation, the supernatants were collected, purified using C18 and GCB sorbents, evaporated under nitrogen, and reconstituted for LC-MS/MS analysis. Hormone separation was performed on a Poroshell 120 EC-C18 column (2.1 × 100 mm, 2.7 μm) with a flow rate of 0.3 mL min^−1^ and an injection volume of 10 μL. The mobile phases consisted of 0.02% formic acid in water (A) and methanol (B), and separation was achieved using a gradient elution program. Quantification was performed using multiple reaction monitoring (MRM) under electrospray ionization (ESI) mode with isotope labeled standards for calibration. Standard curves were generated using mixed hormone standards at concentrations ranging from 0.1 to 200 ng mL^−1^, and all analytes showed good linearity (R^2^ > 0.99). Hormone concentrations were calculated based on the corresponding calibration curves and normalized to fresh weight of tissue. Three independent biological replicates were analyzed for each treatment, with three plants analyzed per treatment in each replicate. Measurements from the plants within each replicate were averaged to obtain one biological replicate for statistical analysis.

Browning of vascular bundles was observed under a stereomicroscope 25 days post inoculation [48]. Approximately 2 cm stem segments from the aboveground tissues were collected, manually cut open, and observed under a stereomicroscope. The extent of vascular browning was evaluated based on visible discoloration of vascular tissues. Five plants per treatment were examined in each independent biological replicate, and the experiment was independently repeated three times.

Fungal colonization was evaluated by fungal recovery assays and qPCR analysis of *V. dahliae* DNA abundance in infected cotton stems. For fungal recovery assays, stem tissues were surface sterilized, cut into 1 cm segments, and placed on Czapek–Dox medium to evaluate fungal recovery. Nine plants per treatment were analyzed in each independent biological replicate, and the experiment was independently repeated three times. For qPCR analysis, genomic DNA was extracted directly from infected stem tissues collected from inoculated plants. Five plants per treatment were analyzed in each independent biological replicate, and the experiment was independently repeated three times. Measurements from the plants within each replicate were averaged to obtain one biological replicate for statistical analysis. The *ITS1* region of *V. dahliae* was amplified using the primer pair described previously, for which specificity to *V. dahliae* was validated in the cited study [49]. The relative abundance of *V. dahliae* DNA was calculated using the 2^−ΔΔCt^ method, with TRV::*00* plants used as the calibrator and cotton *GhUBQ7* used as the reference gene for normalization.

Disease indices were calculated according to previously described methods [50]. Disease severity was evaluated according to the disease rating scale for cotton *Verticillium* wilt. Plants were classified into five grades (0–4) based on symptom severity: grade 0, completely healthy plants without visible symptoms; grade 1, symptoms appearing on one or both cotyledons; grade 2, symptoms appearing on one true leaf; grade 3, symptoms appearing on two or more true leaves; and grade 4, complete wilting of leaves and plant death. The disease index (DI) was calculated using the following formula [51]:

DI = [Σ(number of plants at each disease grade × corresponding disease grade)/(total number of investigated plants × 4)] × 100.

For disease index evaluation, three independent biological replicates were performed, with more than 40 plants analyzed for each treatment in each replicate. The disease index was calculated separately for each replicate, and the resulting three replicate-level values were used for statistical analysis.

### 2.10. Prediction of the GhAOC4/6 Protein–Protein Interaction Network

A predicted protein–protein interaction (PPI) network of GhAOC4/6 was constructed using the STRING database (https://cn.string-db.org/ accessed on 23 March 2026) with a medium confidence threshold (interaction score > 0.4). The filtered interactions were subjected to KEGG pathway and GO functional enrichment analyses to evaluate the functional clustering of candidate interactors.

### 2.11. Data Statistical Analysis

All statistical analyses were performed using GraphPad Prism (version 8.0.2). Data are presented as mean ± standard deviation (SD) from independent biological replicates. The independent biological replicate was used as the statistical unit, and the number of biological replicates (*n*) is indicated in the corresponding figure legends. For comparisons between two groups, an unpaired Student’s *t*-test was used. For the time course RT-qPCR analysis, statistical analyses were performed on ΔCt values using two-way analysis of variance (ANOVA) to evaluate the effects of treatment, time and their interaction, followed by pairwise comparisons between treatments at each time point with Holm correction for multiple comparisons. Relative expression levels in the RT-qPCR analysis were presented as 2^−ΔΔCt^. Statistical significance was defined as *p* < 0.05.

## 3. Results

### 3.1. Identification of the GhAOC Gene Family

Plant allene oxide cyclase, a critical enzyme in the JA biosynthetic pathway, plays essential roles in regulating plant defense responses and growth and development [23,52]. To identify *AOC* gene family members in *G. hirsutum*, a Hidden Markov Model (HMM) was used in combination with BLASTP searches against the *G. hirsutum* genome. After confirmation with the Conserved Domain Database (CDD), a total of six *AOC* family members were identified. These genes are distributed across chromosomes A08, D08, A12, and D12 and were renamed accordingly (Table S1). Evaluation of the physicochemical properties of GhAOCs revealed that their amino acid sequences ranged from 245 to 254 residues in length, with an average molecular weight of approximately 27.30 kDa. The isoelectric points (pI) ranged from 8.51 and 8.95. The instability index of the AOC family proteins ranged from 40.72 to 46.07 and was greater than 40 for all members, predicting that these proteins were unstable in vitro. Meanwhile, the aliphatic index ranged from 72.95 to 83.70, indicating a relatively high content of hydrophobic amino acids and suggesting potential thermostability. Subcellular localization predictions indicated that all GhAOCs were localized to chloroplasts (Table S1). These results indicated that the six AOC proteins in *G. hirsutum* were highly conserved and shared similar physicochemical characteristics and predicted subcellular localization.

### 3.2. Evolutionary Conservation and Diversification of the AOC Gene Family Across Diverse Plant Lineages

A total of 92 members of the AOC family were identified from 26 plant species (Tables S2 and S3). The evolutionary patterns of the AOC protein family across diverse plant lineages were investigated using comparative genomics and molecular evolutionary analyses. The results showed that plant AOC proteins are clearly divided into four major clades (Figure 1A). Among them, Clade III primarily comprises members from early diverging plant lineages, including algae, bryophytes, and pteridophytes (Figure 1A,B). This distribution reflects the presence of AOC homologs in these early diverging lineages. Clades I and II are dominated by multiple AOC homologs from dicot species (Figure 1B). This distribution indicates lineage specific diversification of AOC genes during dicot evolution. Clade IV contains members from monocots, gymnosperms, and several dicot species (Figure 1A,B). This suggests that members of this clade have been conserved across diverse plant lineages. Statistical analysis of AOC protein length distributions revealed greater variation in Clade III than in the other clades. Proteins in the other clades were predominantly 245~255 aa in length (Figure 1C). This pattern suggests that AOC proteins may have been subjected to evolutionary constraints that maintained a relatively conserved protein length during plant evolution. These results indicate that the *AOC* family exhibits substantial evolutionary conservation while undergoing lineage specific diversification during plant evolution.

### 3.3. Evolutionary Conservation and Expansion Mechanisms of the AOC Gene Family in Cotton

To further investigate the evolutionary relationships and conservation of the *AOC* gene family in dicots, a collinearity analysis was conducted using ten representative angiosperm species, including *Arabidopsis thaliana*, *Glycine max*, *Arachis hypogaea*, *Brassica napus*, *Nicotiana tabacum*, *Capsicum annuum*, *Gossypium raimondii*, *Gossypium arboreum*, *Gossypium hirsutum* and *Gossypium barbadense*. Strong synteny was observed among *AOC* genes across the examined species, particularly within the Fabaceae, Brassicaceae, and Malvaceae families (Figure 2A). Notably, syntenic counterparts of *GhAOC4/6* were identified in eight of the ten species (Table S4 and Figure 2A), and most of these counterparts belonged to Clade IV (Figure 1A). These results indicate that Clade IV *AOC* genes, including *GhAOC4/6*, have been conserved during dicot evolution.

Intraspecific synteny analysis was further performed in *Gossypium* species. Two pairs of homologous genes were identified in each of *G. hirsutum* and *G. barbadense*, located on chromosomes A08/D08 and A12/D12, respectively, whereas no homologous gene pairs were detected in *G. arboreum* or *G. raimondii* (Figure S1). Chromosomal localization and duplication type analysis revealed that AOC family members were generated through multiple duplication mechanisms, including tandem duplication (TD), dispersed duplication, whole genome duplication (WGD), segmental duplication, and proximal duplication, as classified using TBtools (version 2.454) (Figure S2 and Table S5). In diploid progenitors *G. arboreum* and *G. raimondii*, TD was the predominant duplication type, accounting for 80% of identified duplication events, with the remaining 20% attributed to dispersed duplication (Figure S3). In contrast, WGD/segmental duplication accounted for 67% of the *AOC* duplication events, whereas TD accounted for 33%. In *G. barbadense*, WGD/segmental duplication accounted for 45% of the duplication events, while TD accounted for 33% (Figure S3). Thus, WGD/segmental duplication contributed substantially to *AOC* gene family expansion in allotetraploid cotton. These results indicate that different duplication mechanisms contributed to *AOC* gene family expansion at different stages of cotton evolution. TD contributed predominantly to expansion in diploid progenitors, whereas WGD/segmental duplication became more prominent in allotetraploid cotton.

Ka/Ks analysis further showed that AOC gene pairs within four Gossypium species were predominantly subjected to purifying selection (Table S6). Gene pairs with Ka = 0 and Ks = 0 were recorded as zero-substitution pairs and were considered completely conserved, without assigning a numerical Ka/Ks value because the ratio was undefined. These results suggest that duplicated AOC genes have been evolutionarily constrained following gene duplication. The widespread purifying selection indicates that many duplicated AOC copies have been preferentially retained during cotton evolution, although whether these genes have undergone functional divergence requires further investigation.

### 3.4. Conserved Domain and N-Terminal Variation Underlie AOC Functional Diversity in Cotton

A phylogenetic tree was constructed for 25 *AOC* gene members from *G. raimondii*, *G. arboreum*, *G. hirsutum* and *G. barbadense* (Figure 3A). Their conserved domains, motifs, and gene structures were subsequently analyzed. Each AOC protein contains the conserved PLN02343 domain, which belongs to the Allene_ox_cyc superfamily (Figure 3A,D). Further motif analysis identified eight conserved sequence motifs in cotton AOC proteins (Figure 3B). Motif 1 contains the highly conserved active site region [KVQEL(F/L/S)VYEINERDRG] of the PLN02343 domain. It also contains the region involved in substrate binding and catalysis [GDLVPF(S/T)NK(L/I/Y)] (Figure S4). These conserved regions contain residues essential for AOC catalytic activity and are therefore likely important for JA synthesis. The main differences among cotton AOC proteins were observed in their N-terminal regions. These differences were associated mainly with the presence or absence of N-terminal motifs and the arrangement, whereas the motif sequences within the conserved regions were highly similar (Figure 3B). In terms of gene structure, most *AOC* genes exhibited similar exon-intron organization, with two or three exons. However, several members exhibit distinct gene structures. For example, *GbAOC8* lacks introns, whereas *GhAOC2* and *GhAOC5*contain particularly long introns (Figure 3C). The three-dimensional structures of AOC proteins from cotton and *Arabidopsis* were predicted using AlphaFold. The predicted structures showed strong conservation within the Allene_ox_cyc domain, which contains eight β-strands (Figure 3E and Figure S5). In contrast, structural variations were mainly observed in the irregular coiled-coil region formed by the N-terminal domain. Together, these results indicate that the core functional domain of cotton AOC proteins is highly conserved, whereas the N-terminal region exhibits greater structural variation. This structural variation may contribute to functional differentiation among AOC family members.

### 3.5. Potential Transcriptional Regulatory Features of Cotton AOC Genes

*Cis*-acting elements in promoter regions are important regulatory features associated with gene expression. The 1500 bp upstream regions of *AOC* genes from *Gossypium* species were analyzed using the PlantCARE database. The results showed that *AOC* promoters contained various predicted *cis*-acting elements associated with light responsiveness, stress responses, and hormone responsiveness, including MeJA, abscisic acid (ABA), gibberellin (GA), auxin (IAA), and salicylic acid (SA) responses (Figure 4A and Table S7). The number and composition of predicted *cis*-elements varied among different *AOC* members, suggesting that *AOC* genes may possess diverse promoter architectures and potentially exhibit distinct transcriptional regulation patterns. In addition to numerous light responsive elements, *AOC* promoters were enriched in hormone responsive elements, particularly those associated with ABA and MeJA responses (Figure 4B). These patterns indicate that *AOC* promoters contain diverse predicted regulatory features. Notably, MeJA responsive elements were detected in most *AOC* members, whereas these elements were not identified in the promoters of *Gossypium AOC* members belonging to Clade IV (Figure 1A and Figure 4B). This difference indicates that the predicted promoter architectures of Clade IV members differ from those of other *AOC* genes. However, the presence or absence of predicted *cis*-elements does not by itself establish hormone responsive transcriptional regulation. Functional assays will be required to determine whether these predicted elements contribute to the regulation of individual *AOC* genes.

Further prediction of transcription factor binding sites revealed potential binding sites for 15 transcription factor families in *AOC* promoters, including AP2, MYB, bHLH, NAC, ERF, GATA, bZIP, C2H2, Dof, Trihelix, TCP, TALE, Nin-like, and BBR-BPC (Figure 4C and Table S8). Among these, predicted binding sites for bZIP, bHLH, and Dof were widely distributed and occurred at relatively high frequencies in multiple *AOC* promoters (Figure 4D). KEGG and GO enrichment analyses of these predicted transcription factors showed enrichment in pathways associated with plant hormone signaling and transcriptional regulation (Figure S6). These results provide candidate regulatory features and potential transcription factors for further investigation, but they do not establish direct transcription factor binding or transcriptional regulation of *AOC* genes.

### 3.6. Expression Profiles of GhAOC Genes Across Tissues and Under Stress Conditions

To investigate the expression profiles of *GhAOC* genes across different tissues and in response to stress, available RNA-seq data were analyzed (Table S9). *GhAOC1* exhibited the highest overall expression level in all tissues examined, whereas *GhAOC3* showed extremely low expression in most tissues. Compared with other tissues, the expression of *GhAOC* genes was negligible in petals and stamens, whereas higher expression levels were observed in roots (Figure 5A). Examination of expression patterns during ovule development showed that *GhAOC1* and *GhAOC2* were highly expressed in −1 DPA ovules (Figure 5B), suggesting an association with the early stage of fiber initiation. During the fiber elongation stage, *GhAOC4* exhibited elevated expression. During the secondary wall thickening stage, the expression of *GhAOC1* and *GhAOC2* increased (Figure 5C). These expression patterns suggest that these genes may be associated with different stages of fiber development.

All members of the *GhAOC* family responded to abiotic stress treatments, with *GhAOC1* and *GhAOC2* showing relatively stronger induction under several stress conditions (Figure 5D–G). In response to cold stress, the expression of *GhAOC1*, *GhAOC2*, and *GhAOC5* increased rapidly, whereas *GhAOC6* showed a gradual increase (Figure 5D). Under heat stress, all members except *GhAOC3* exhibited increased expression within one hour (Figure 5E). Under PEG and NaCl stress, *GhAOC1/2/4/5* displayed relatively stronger responses (Figure 5F,G). When resistant and susceptible cotton cultivars were inoculated with *V. dahliae*, *GhAOC4* and *GhAOC6* exhibited stronger pathogen responsive expression compared with other *GhAOCs* (Figure 5H).

To further characterize their responses to *V. dahliae* infection, root samples were collected at 0, 6, 12, 24, 48, and 72 h after inoculation for RT-qPCR analysis. *GhAOC6* expression was significantly induced at 6, 12, 24, and 72 h post inoculation compared with the control (Figure 5I), indicating a sustained response during infection. *GhAOC1*, *GhAOC2*, *GhAOC3*, *GhAOC4* and *GhAOC5* also showed induction at specific time points (Figure 5I).

### 3.7. Subcellular Localization of GhAOCs

To determine the subcellular localization of GhAOCs, the full-length coding sequences of GhAOCs were fused to the N-terminus of eGFP and transiently expressed in *Nicotiana benthamiana* leaves. The empty vector control expressing eGFP (CK) showed fluorescence mainly in the nucleus and cytoplasm, whereas GhAOC1-eGFP and GhAOC2-eGFP fluorescence was exclusively detected in chloroplasts (Figure 6). In contrast, GhAOC3-eGFP, GhAOC4-eGFP, GhAOC5-eGFP and GhAOC6-eGFP showed fluorescence in both chloroplasts and at the cell periphery. Chloroplast autofluorescence overlapped with the fluorescence signals of all GhAOC fusion proteins, supporting their chloroplast localization. To further verify the cell periphery localization of GhAOC3/4/5/6, the plasma membrane marker AtPIP2A-mCherry was co-expressed with GhAOC3/4/5/6-eGFP. The fluorescence signals of GhAOC3/4/5/6-eGFP at the cell periphery overlapped with the AtPIP2A signal. In addition, plasmolysis was performed for both the CK and GhAOC3/4/5/6-eGFP constructs. Following plasmolysis, the plasma membrane associated fluorescence of GhAOC3/4/5/6-eGFP were observed along the retracted plasma membrane (Figure 6), further supporting preliminary plasma membrane localization. But the preliminary plasma membrane localization of GhAOC3/4/5/6 requires further validation by biochemical fractionation in future studies.

### 3.8. Silencing of GhAOC4/6 Reduces Cotton Resistance to Verticillium Wilt

Based on the quantitative expression analysis, *GhAOC6* showed the most pronounced transcriptional response to *V. dahliae* infection among the *GhAOC* members examined, therefore selected as the initial candidate for functional analysis. However, sequence alignment revealed that the CDS sequences of *GhAOC4* and *GhAOC6* were nearly identical, differing only by a three-nucleotide deletion (Figure S7 and Table S10). Given this high sequence similarity and the inability of the VIGS system to reliably distinguish between these two homologous genes, *GhAOC4/6* were therefore selected as a gene pair for subsequent functional analysis. A single *GhAOC4/6* shared fragment was used to generate the VIGS construct (Figure S7). Therefore, *GhAOC4* and *GhAOC6* were simultaneously silenced by VIGS. The effectiveness of the VIGS system was confirmed by silencing *PDS,* which resulted in obvious leaf bleaching phenotypes seven days after injection (Figure 7A). RT-qPCR analysis showed that the transcript levels of *GhAOC4* and *GhAOC6* were reduced by approximately 40.34% and 46.54%, respectively, in the corresponding silenced plants compared with the TRV::*00* control (Figure 7B). To assess the specificity of *GhAOC4/6* silencing, the transcript levels of the remaining *GhAOC* members were also examined in the VIGS plants. The expression levels of *GhAOC1*, *GhAOC2*, *GhAOC3*, and *GhAOC5* fluctuated by approximately 5% to 12% compared with the control plants, and none of these changes was statistically significant (Figure S8A). In contrast, *GhAOC4* and *GhAOC6* expression was significantly reduced by approximately 40.3% and 46.5%, respectively. These results indicate that the VIGS treatment predominantly affected *GhAOC4* and *GhAOC6*. The expression levels of the defense and JA responsive genes *GhHIN1*, *GhHSR203*, *GhRBOHD*, *GhMYC2* and *GhPR4* were significantly reduced in the *GhAOC4/6* silenced plants compared with the control plants (Figure S8B). These results suggest that *GhAOC4/6* silencing is associated with reduced expression of defense related genes and impaired plant defense responses.

Following inoculation with *V. dahliae*, the second true leaves were collected at 24 h post inoculation for DAB staining. DAB staining revealed weaker H_2_O_2_ associated staining in *GhAOC4/6* silenced plants than in control plants (Figure 7C). Furthermore, at 72 h post inoculation, phloroglucinol staining also showed weaker lignin associated staining in the *GhAOC4/6* silenced plants (Figure 7D). These observations indicate that *GhAOC4/6* co-silencing was associated with weaker H_2_O_2_ and lignin staining during the early stages of infection. Disease progression was evaluated at 25 days post inoculation. Compared with TRV::*00* plants, *GhAOC4/6* silenced plants displayed more severe disease symptoms, including increased leaf yellowing and abscission (Figure 7E). The disease index increased by approximately 20% after suppression of *GhAOC4/6* (Table S11 and Figure 7F). In addition, vascular browning was enhanced in silenced plants (Figure 7G). Fungal recovery assays revealed higher fungal recovery from the stems of *GhAOC4/6* silenced plants than from control plants (Figure 7H). Consistently, qPCR analysis showed a higher relative abundance of *V. dahliae* DNA in the stems of *GhAOC4/6* silenced plants (Figure 7I). Collectively, these results indicated that reduced expression of *GhAOC4/6* compromises cotton resistance to *V. dahliae*.

Because AOC catalyzes the formation of 12-OPDA, a precursor of downstream JA, the levels of 12-OPDA, JA, and JA-Ile were further quantified. Cotton stem samples were collected at 15 days post inoculation, a time point selected to evaluate the overall impact of *GhAOC4/6* suppression on JA metabolite accumulation during disease development. Previous studies have shown that JA metabolites undergo dynamic changes after pathogen infection and contribute to the regulation of defense responses at different stages of infection. After establishment of standard curves, the concentrations of these compounds were quantified by UPLC-MS/MS analysis. Compared with TRV::*00* plants, *GhAOC4/6* silenced plants significantly reduced 12-OPDA levels by 37.27% (Figure 7J). The levels of JA decreased by 79.87% (Table S11 and Figure 7K), while JA-Ile, the bioactive conjugated form responsible for activating JA signaling decreased by 61.28%. These results demonstrated that *GhAOC4/6* silenced plants exhibited lower levels of JA related metabolites than control plants following *V. dahliae* infection.

### 3.9. Predicted Protein Interaction Network of GhAOC4/6

To further explore potential molecular association related to GhAOC4/6, a protein interaction network was predicted using the STRING database (Figure 8A). KEGG and GO enrichment analyses were performed on the proteins predicted to be associated withGhAOC4/6 (Table S12). KEGG enrichment analysis showed that these predicted proteins were significantly enriched in fatty acid metabolism pathways, such as α-linolenic acid metabolism and fatty acid degradation/metabolism (Figure 8B). These enrichment patters suggest that GhAOC4/6 may be associated with biological processes related to lipid homeostasis and fatty acid turnover. Notably, α-linolenic acid metabolism is the primary route for JA biosynthesis. Moreover, KEGG enrichment also identified hormone signal transduction pathways, indicating that the predicted GhAOC4/6 interaction proteins may be connected to hormone related processes.

GO enrichment analysis further showed that the predicted GhAOC4/6 associated proteins were mainly enriched in chloroplast and peroxisome related cellular components, consistent with the subcellular localization of GhAOC4/6 (Figure 6 and Figure 8C). The enriched biological processes were primarily related to lipid metabolic processes, in agreement with the KEGG enrichment results. The molecular function categories included oxidoreductase activity, lipid binding, and DNA binding transcription factor activity. Overall, these computational predictions suggest potential functional associations between GhAOC4/6 and proteins involved in lipid metabolism and hormone-related processes. While these predicted protein associations remain experimentally unverified, they offer valuable avenues for future investigation.

## 4. Discussion

AOC has been widely studied as a key enzyme in JA biosynthesis, and previous functional studies have demonstrated that individual *AOC* genes can influence plant responses to pathogens, insects, and abiotic stresses in several species [19,21,33]. These studies have established the importance of AOC mediated JA biosynthesis in plant adaptation but have largely focused on individual genes or specific physiological processes. In contrast, the present study provides an integrated analysis of the *AOC* gene family across representative *Gossypium* species and links family level evolutionary diversification with pathogen responsive functional differentiation in cotton. Our comparative analyses revealed strong conservation of the AOC catalytic domain together with diversification in gene structure, N-terminal regions, promoter architecture, and expression patterns. Importantly, the pathogen responsive *GhAOC6* and *GhAOC4* were identified as candidate components associated with cotton resistance to *V. dahliae*. Co-silencing of *GhAOC4/6* compromised cotton resistance to *V. dahliae*, and changes were observed in weaker H_2_O_2_ staining, lignin staining and JA related metabolites at different stages of infection. These findings extend previous studies of individual *AOC* genes by providing a systematic evolutionary characterization of the *AOC* gene family in cotton and identifying *GhAOC4/6* as candidate component associated with cotton resistance to *V. dahliae*.

Comparative evolutionary analysis showed that AOC proteins are broadly conserved across plant lineages, while also exhibiting lineage specific diversification. Phylogenetic analysis classified plant AOCs into four major clades, with cotton AOCs mainly distributed in Clade II and Clade IV (Figure 1). Interestingly, dicotyledonous AOCs were distributed among all four clades, whereas the monocotyledonous species analyzed in this study were only found in Clade IV. However, because only representative monocot genomes were included, it remains unclear whether other monocot lineages contain AOC members belonging to additional clades. More comprehensive genome wide surveys across diverse monocot species will be required to resolve this evolutionary pattern. In addition, AOCs from early diverging plant lineages displayed greater variation in protein length, whereas gymnosperm and angiosperm AOCs showed relatively conserved sizes (Figure 1). Rather than representing convergent evolution, this pattern more likely reflects evolutionary constraints maintaining the structural requirements of AOC proteins. Similar evolutionary conservation has also been observed in other conserved metabolic enzymes, such as CPS in the gibberellin biosynthetic pathway [53], suggesting that enzymes involved in essential hormone metabolism may be subjected to strong structural constraints.

Gene duplication analysis indicated that different evolutionary mechanisms contributed to *AOC* family expansion in cotton. Tandem duplication contributed to *AOC* expansion in diploid cotton species, whereas whole genome duplication and segmental duplication became more prominent following polyploidization. The strong purifying selection detected among duplicated *AOC* genes suggests that retained copies have experienced functional constraints during evolution, possibly reflecting the importance of maintaining conserved JA-associated metabolic functions. Although most cotton *AOCs* exhibited similar exon-intron structures, several structural variations were observed, including long introns in *GhAOC2* and *GhAOC5* and intron loss in *GbAOC8* (Figure 3). Such structural divergence may influence transcript processing, expression regulation, or protein function [54,55], thereby providing potential sources for functional diversification among *AOC* members.

Promoter analysis further suggested that cotton AOCs may be integrated into complex regulatory networks involving environmental and hormonal signals. The enrichment of light, hormone, and stress responsive *cis*-elements indicates that *AOC* expression may respond to diverse developmental and environmental cues. Notably, MeJA responsive elements were absent from the promoters of Clade IV members, including *GhAOC4* and *GhAOC6* (Figure 4). This difference may reflect divergence in promoter architecture during cotton evolution. However, the functional significance of these predicted elements remains unknown and requires experimental validation. In addition, multiple transcription factor binding sites, particularly those associated with bZIP, bHLH, NAC, and AP2/ERF families, were predicted in AOC promoters. Previous studies have shown that certain transcription factors, such as ERF026 [56] and bZIP72 [57], can directly regulate *AOC* expression in other plant species, suggesting that their cotton homologs may represent potential candidates for future investigation of *GhAOC* transcriptional regulation.

All GhAOC proteins were predicted to be localized to the chloroplast, which is consistent with previous studies of AOC subcellular localization in other plant species and further supports the classical view that JA biosynthesis begins in chloroplasts [58]. The additional preliminary plasma membrane associated fluorescence was supported by co-localization with AtPIP2A and plasmolysis. However, because these observations were obtained using transient expression of GFP tagged proteins, the possibility that protein abundance or the GFP fusion itself influenced the peripheral fluorescence cannot be completely excluded.

Expression profiling revealed functional differentiation among cotton *AOC* members. The broad expression pattern of some members suggests potential involvement in fundamental physiological processes, whereas the tissue preferential expression of others indicates possible specialization (Figure 5). The relatively high expression of *GhAOC4* during early fiber development suggests a potential association with fiber developmental regulation, which is consistent with recent QTL evidence linking *GmAOC4* to fiber related traits [34]. JA treatment has been shown to promote secondary wall synthesis [59]. The elevated expression of *GhAOC1* and *GhAOC2* during the secondary wall thickening stage suggests that these genes may be associated with secondary wall development in cotton fibers. The preferential expression of *GhAOC1/2/4/6* in roots suggests potential roles in root development and defense, given that roots play central roles in water and nutrient acquisition while also serving as the primary barrier against soil borne pathogens [60]. To investigate the contribution of *AOC* members to cotton resistance against *V. dahliae*, *GhAOC6* was selected as candidate gene based on expression patterns, evolutionary conservation, and potential relevance to stress responses. However, because *GhAOC4* and *GhAOC6* are homologous genes, VIGS mediated silencing strategy could not distinguish between the two genes. Therefore, the results reflect the combined contribution of the *GhAOC4/6* genes rather than the independent function of each gene. Future studies using CRISPR based gene editing, together with overexpression and complementation approaches, will be necessary to distinguish the individual roles and potential functional redundancy of these highly similar members. Co-silencing of *GhAOC4/6* increased cotton susceptibility to Verticillium wilt. The silenced plants showed weaker H_2_O_2_ and lignin staining at 24 and 72 h post inoculation, respectively, together with lower levels of JA related metabolites at 15 days post inoculation. Because these indicators were measured at different stages of infection, these results do not establish a temporal or causal relationship among *GhAOC4/6*, JA metabolism, H_2_O_2_ and lignin responses. Further time course experiments are needed to clarify their relationships during infection. Previous studies have shown that exogenous application of MeJA can enhance cotton resistance to *V. dahliae*, supporting an important role of JA-associated responses in cotton *Verticillium* wilt resistance [61,62]. In the future, further comparative studies using resistant and susceptible cotton genotypes, together with individual functional analyses of *GhAOC4* and *GhAOC6*, will be necessary to determine their genotype dependent contributions to *Verticillium* wilt resistance. The predicted protein interaction network serves as a basis for future research by identifying candidate proteins and pathways potentially associated with GhAOC4/6 (Figure 8). However, since these associations are based solely on computational predictions, they require further experimental validation.

## 5. Conclusions

In this study, the *AOC* gene family was identified and systematically characterized across four representative *Gossypium* species, including *G. hirsutum*, *G. barbadense*, *G. arboreum* and *G. raimondii*. Phylogenetic analysis revealed that cotton AOCs were mainly distributed in Clade II and Clade IV. Synteny analysis showed that TD was the major driving force underlying AOC family expansion in the diploid cotton species *G. arboreum* and *G. raimondii*, whereas WGD/segmental duplication was the predominant contributor in the tetraploid cotton species *G. hirsutum* and *G. barbadense*. Structural and promoter analyses indicated that AOCs possess conserved Allene_ox_cyc domains but exhibit variation in gene structure and regulatory elements, suggesting potential functional specialization among family members. Expression profiling in *G. hirsutum* revealed distinct tissue specific, developmental and pathogen responsive patterns, identifying *GhAOC6* as candidate genes associated with cotton responses to *V. dahliae* infection. Due to their high sequence similarity, *GhAOC4* and *GhAOC6* could not be individually distinguished by the VIGS system and were therefore considered as a candidate gene pair. Subcellular localization analysis showed that GhAOC4 and GhAOC6 were localized to chloroplasts, suggesting their potential involvement in multiple cellular processes related to JA-associated defense responses. Functional analysis using VIGS demonstrated that co-silencing of *GhAOC4*/*6* compromised cotton resistance to *V. dahliae*, accompanied by weaker H_2_O_2_ and lignin staining and altered levels of JA related metabolites. These findings indicate that *GhAOC4/6* co-silencing was associated with reduced cotton resistance to *Verticillium* wilt. Further studies using individual overexpression and CRISPR based gene editing of *GhAOC4* and *GhAOC6* will be required to distinguish their individual functions. Collectively, this study provides a systematic characterization of the *AOC* gene family in *Gossypium* and identifies *GhAOC4/6* as candidate components contributing to cotton resistance to *V. dahliae*.

## Figures and Tables

**Figure 1:**
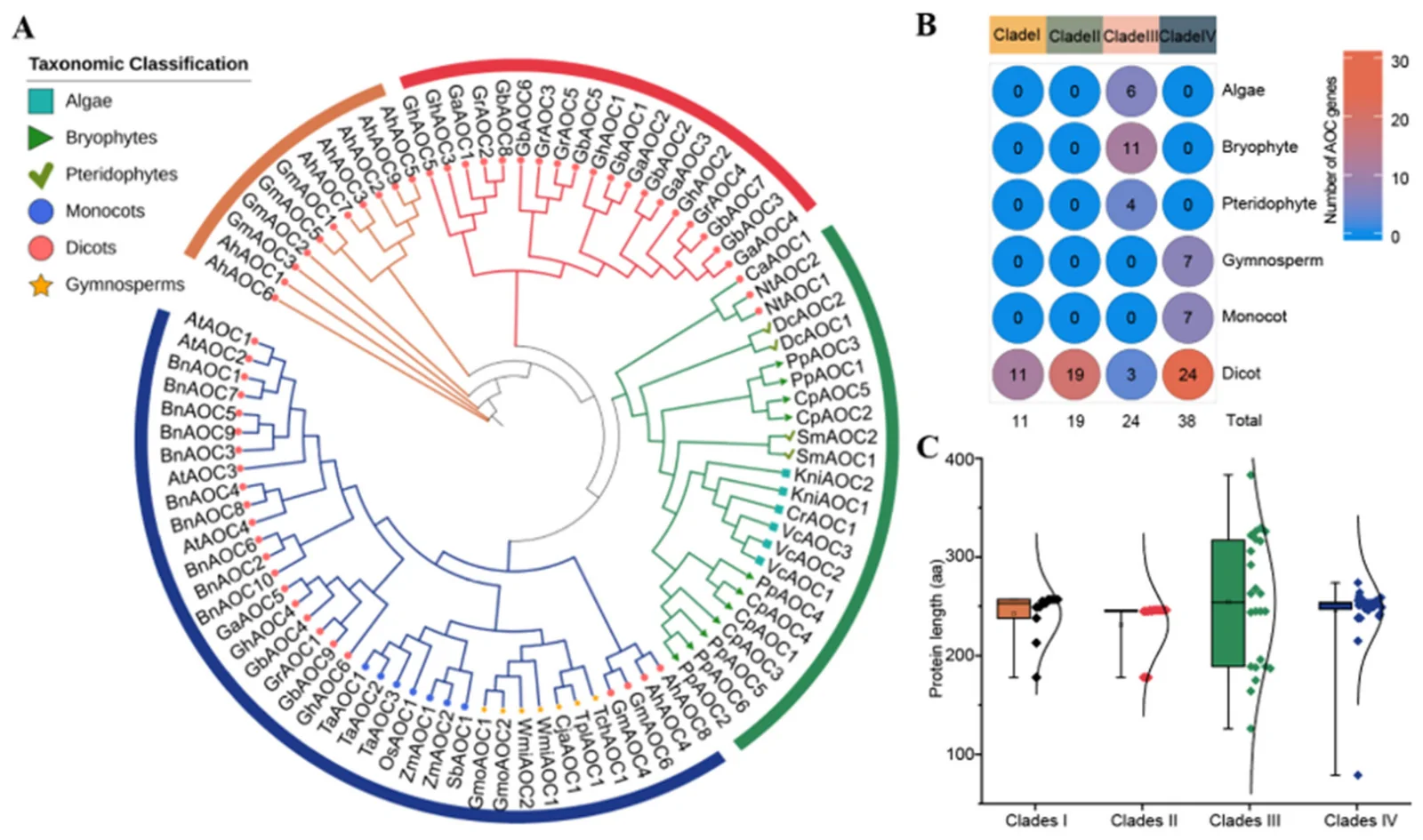
Phylogenetic tree of AOC proteins. (**A**) Phylogenetic tree of the *AOC* gene family across 26 representative plant species. The four subfamilies (clades I–IV) are color coded. The phylogenetic tree is constructed using IQ-TREE with the Q.PFAM + G4 amino acid substitution model and 5000 bootstrap replicates and was rooted using midpoint rooting. Taxa are indicated as follows: Algae: square; Bryophytes: triangle; Pteridophytes: check mark; Monocots: blue circle; Dicots: red circle; Gymnosperms: star. (**B**) Number of AOC members in different phyla across different clades. (**C**) Distribution of protein amino acid lengths among members of different clades.

**Figure 2:**
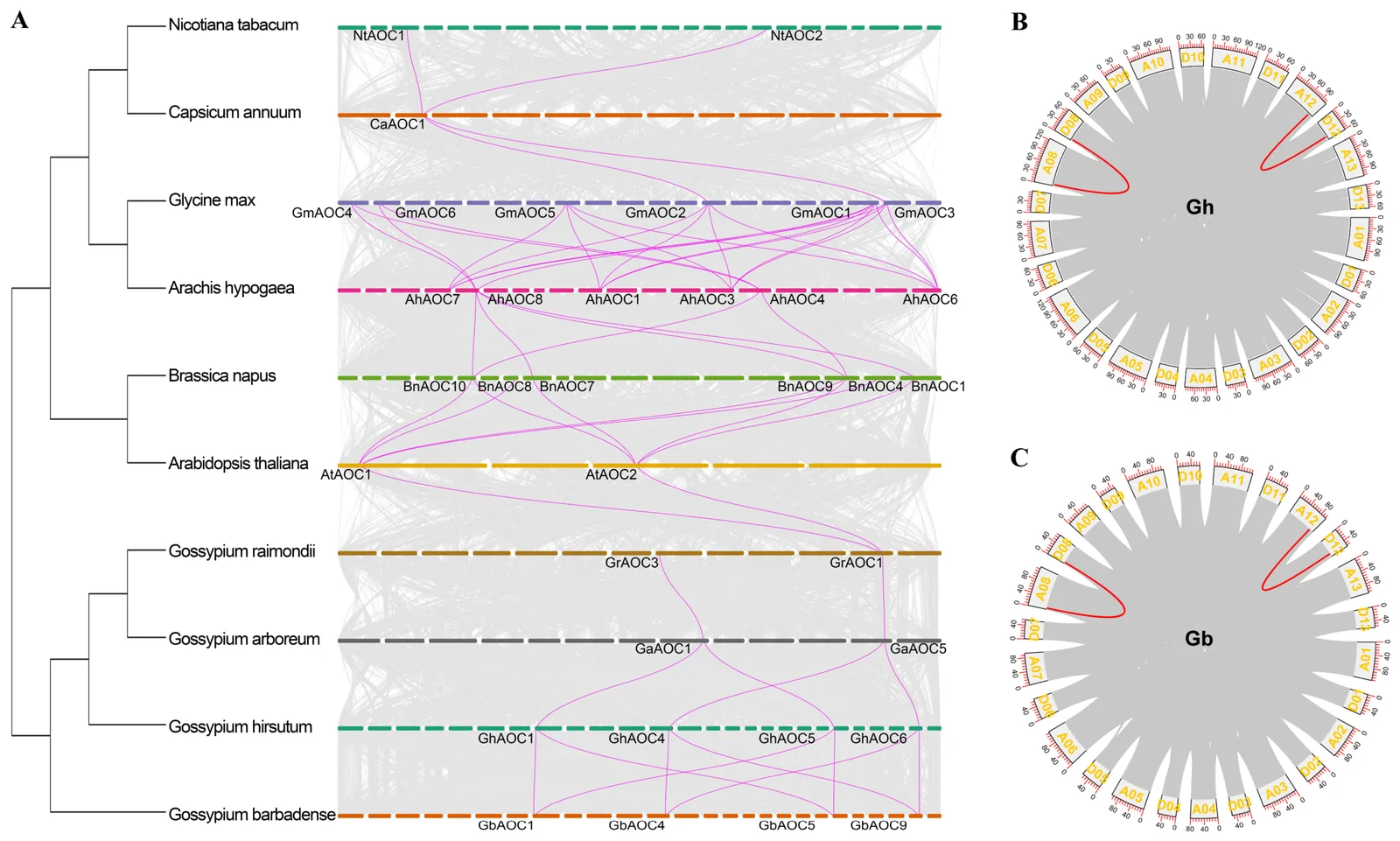
Interspecific and intraspecific synteny analysis of AOC genes. (**A**) Interspecific synteny analysis of AOC genes among ten dicot species. (**B**,**C**) Intraspecific synteny analysis of AOC genes in *Gossypium hirsutum* (**B**) and *Gossypium barbadense* (**C**). Gray lines indicate syntenic regions between or within species, whereas purple and red lines represent syntenic AOC gene pairs.

**Figure 3:**
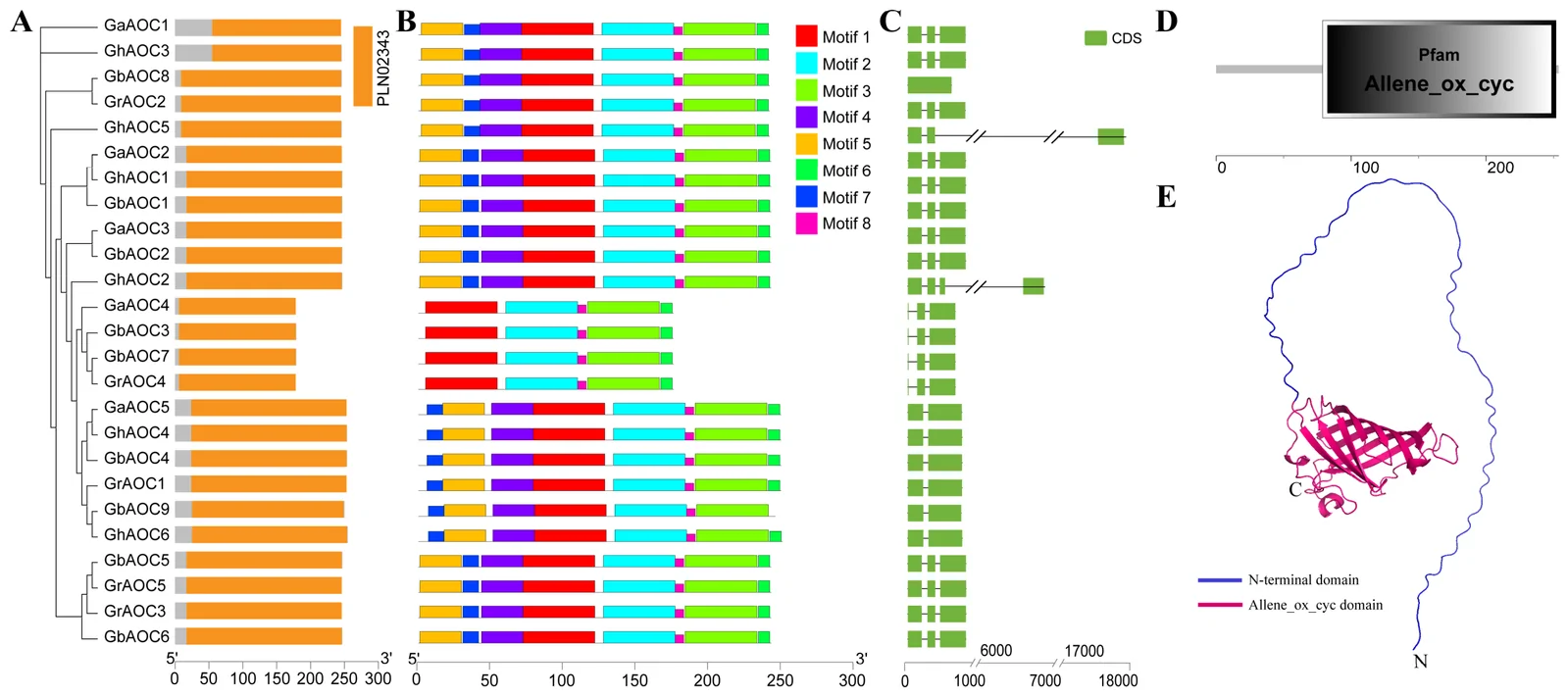
Conserved domains and gene structure of AOC genes in cotton. (**A**) Conserved domains of AOC proteins. (**B**) Motif analysis of AOC proteins. (**C**) Exon-intron structures of AOC genes. (**D**,**E**) Conserved domains and 3D structure of GhAOC6.

**Figure 4:**
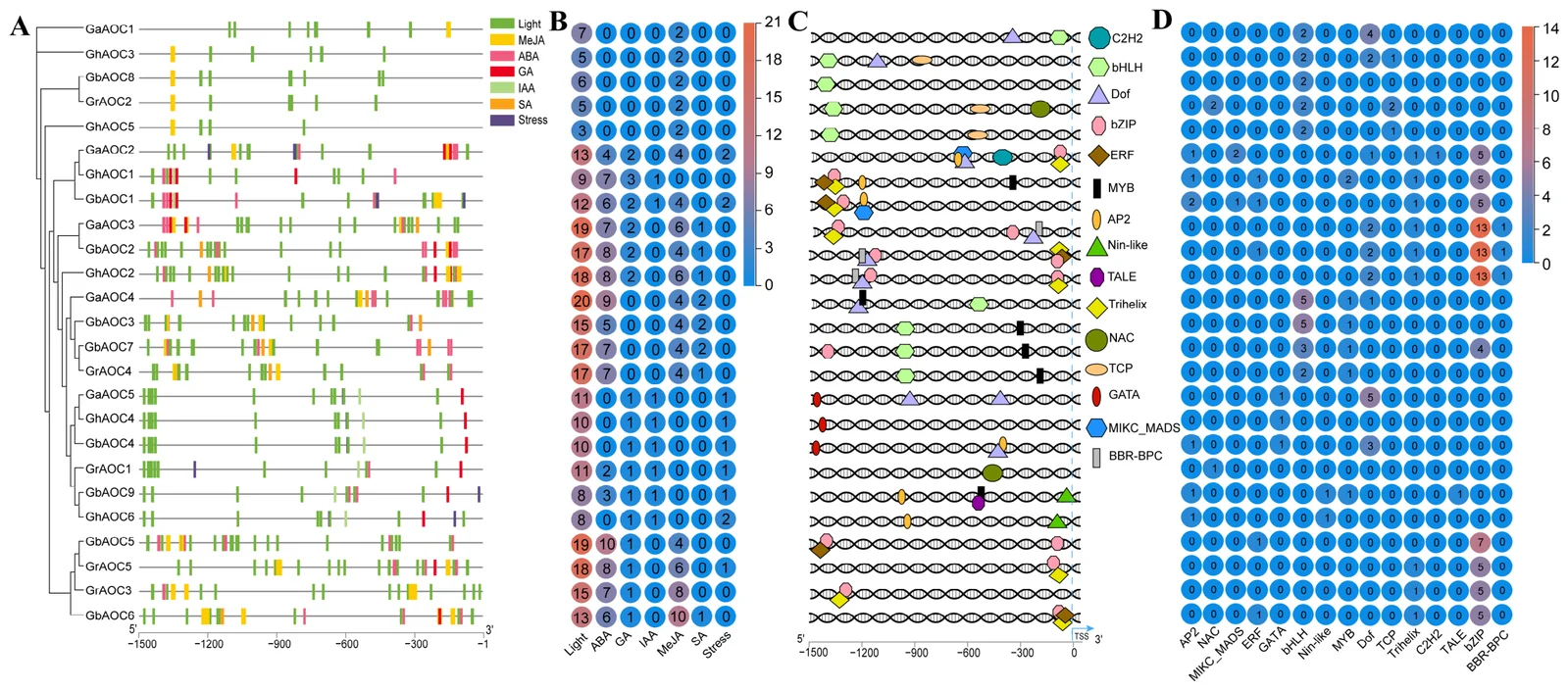
Predicted *cis*-acting elements and transcription factor binding sites in the promoters of *AOC* genes in cotton. (**A**) Predicted *cis*-acting elements in the promoters of *AOC* genes. (**B**) Distribution of *cis*-acting elements across *AOC* members. (**C**) Predicted transcription factor binding sites in the promoters of *AOC* genes. (**D**) Distribution of transcription factor binding sites across *AOC* genes.

**Figure 5:**
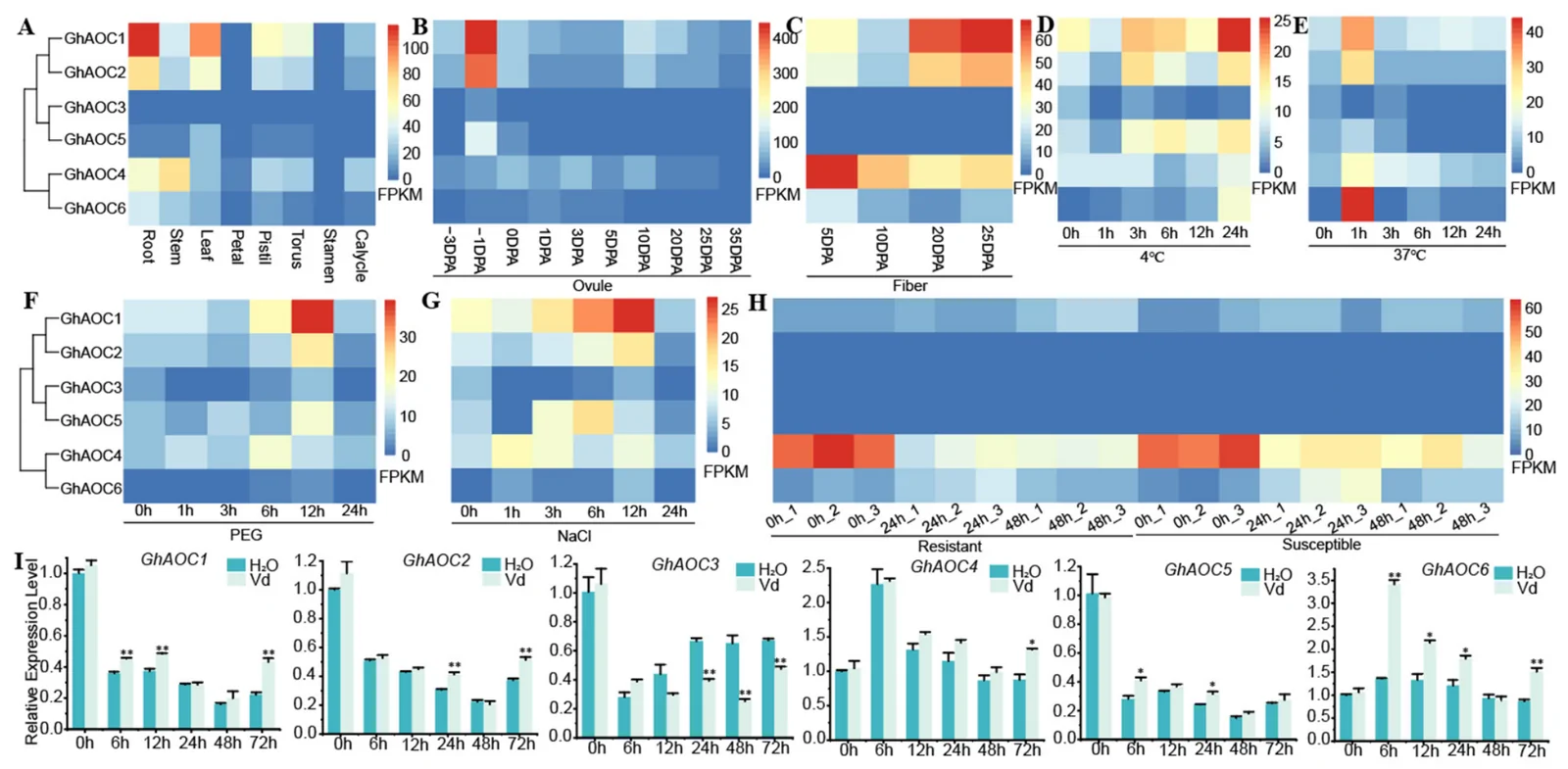
Expression profiles of *GhAOC* genes in different tissues and under various stress conditions. (**A**) Expression profiles of *GhAOC* genes in different cotton tissues. (**B**,**C**) Expression profiles of *GhAOC* genes in cotton ovules (**B**) and fibers (**C**) at different developmental stages. (**D**–**G**) Expression profiles of *GhAOC* genes under cold (**D**), heat (**E**), PEG (**F**) and salt (**G**) stress treatments. (**H**) Expression of *GhAOC* genes in resistant (R) and susceptible (S) cultivars following *Verticillium dahliae* inoculation. (**I**) RT-qPCR validation of the expression patterns of *GhAOC* genes following *Verticillium dahliae* inoculation. For panel (**I**), statistical analyses were performed on ΔCt values using two-way ANOVA, followed by pairwise comparisons between H_2_O and *V. dahliae* treatments at each time point with Holm correction for multiple comparisons. Relative expression levels are presented as 2^−ΔΔCt^. Data are presented as mean ± SD from three independent biological replicates (n = 3). *p* < 0.05 (*) and *p* < 0.01 (**).

**Figure 6:**
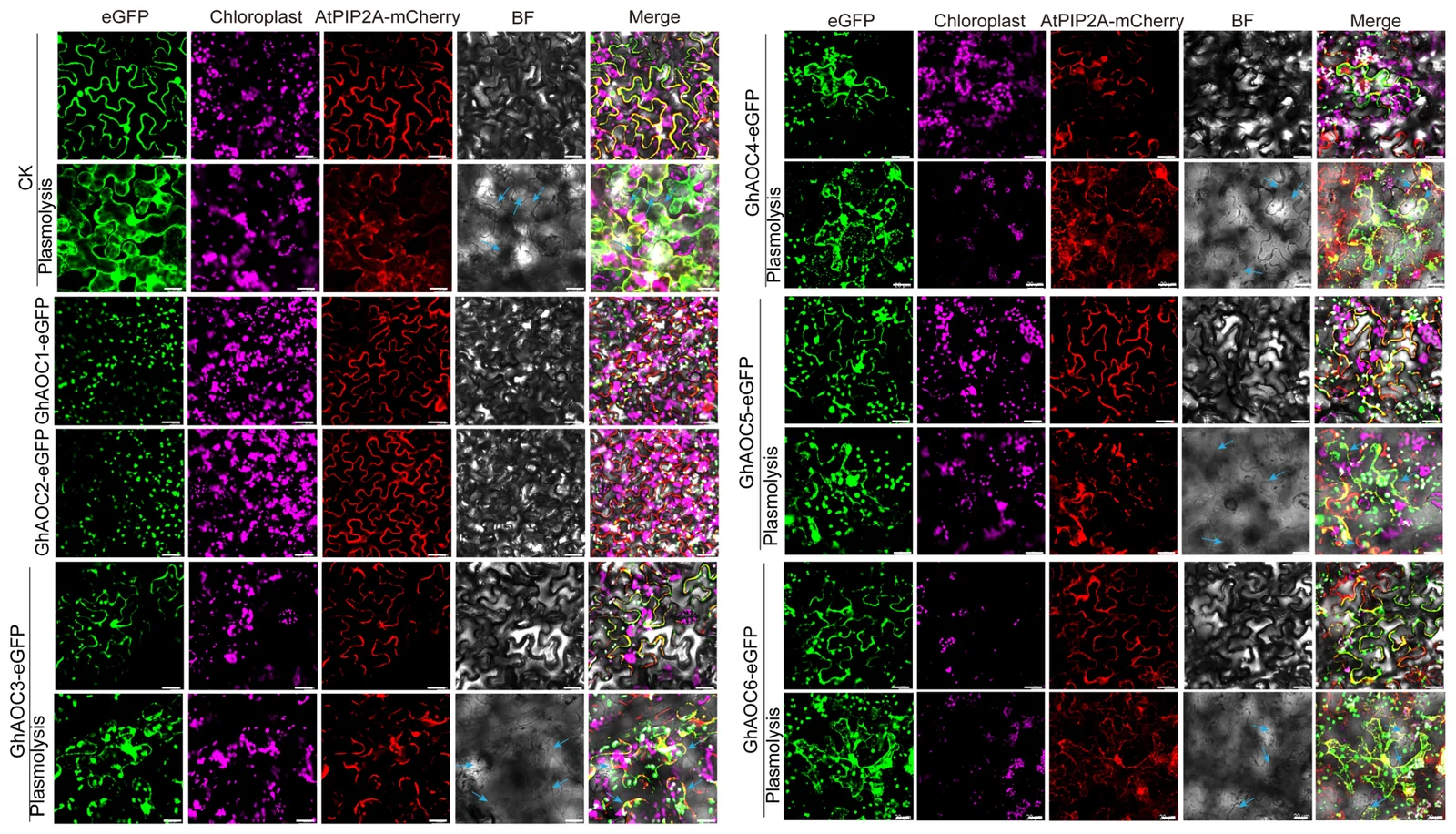
Subcellular localization of GhAOCs. GhAOCs-eGFP was transiently expressed, with an empty vector expressing eGFP used as the control (CK). The plasma membrane marker AtPIP2A was co-expressed to assess plasma membrane associated fluorescence. Plasmolysis was performed by treating tobacco leaves with 0.8 M mannitol. The blue arrows indicated the sites of plasmolysis in tobacco leaf mesophyll cells. Scale bar = 20 μm.

**Figure 7:**
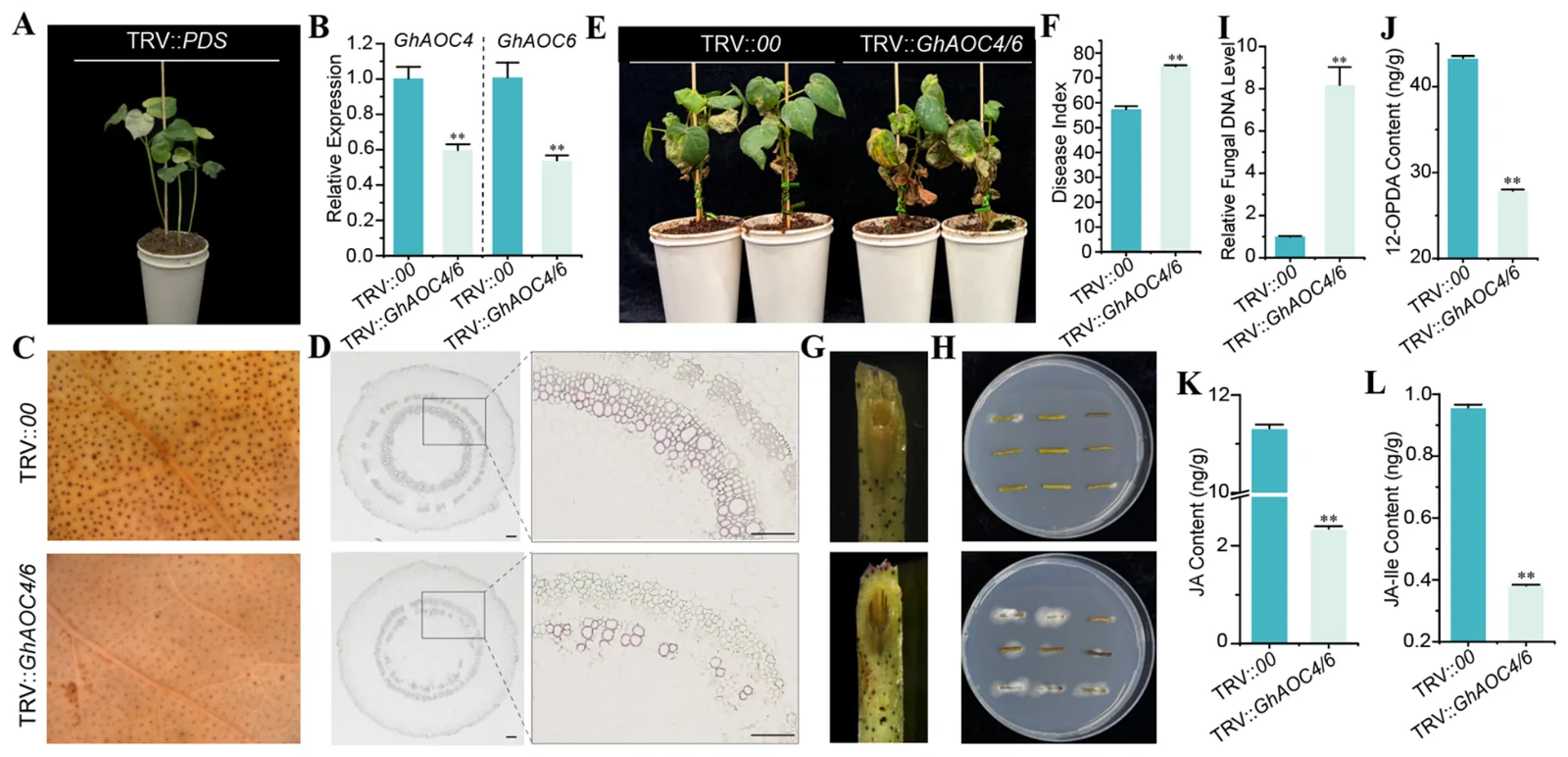
Silencing of the *GhAOC4/6* genes reduces cotton resistance to *Verticillium* wilt. (**A**) TRV::*PDS* induced leaf bleaching is shown as a positive control. (**B**) Silencing efficiencies of *GhAOC4* and *GhAOC6* are assessed by RT-qPCR. (**C**) DAB staining of leaves at 24 h after inoculation with *Verticillium dahliae*. (**D**) Phloroglucinol staining of stems at 72 h after inoculation with *Verticillium dahliae*. (**E**) Disease symptoms of *GhAOC4/6* silenced and control plants after inoculation with *Verticillium dahliae*. (**F**) Disease index of *GhAOC4/6* silenced and control plants. (**G**) Vascular bundle browning in cotton stems at 25 days after inoculation with *Verticillium dahliae*. (**H**) Fungal recovery from *GhAOC4/6* silenced and TRV::*00* control plants at 25 days post inoculation. (**I**) Relative abundance of *Verticillium dahliae* DNA in infected stems was determined by qPCR analysis of the ITS1 region. (**J**–**L**) Silencing of *GhAOC4/6* reduces the levels of 12-OPDA (**J**), JA (**K**), and JA-Ile (**L**) in cotton stems. Three independent biological replicates were performed for each assay. For panels (**B**–**D**), two plants were analyzed per replicate; for panels (**G**,**I**), five plants were analyzed per replicate; for panel (**H**), nine plants were analyzed per replicate; and for panels (**J**–**L**), three plants were analyzed per replicate. For disease index analysis (**F**), more than 40 plants were evaluated for each treatment within each independent replicate, and the disease index was calculated separately for each replicate. The mean ± SD values shown in the quantitative panels were calculated from three independent biological replicates (n = 3). Statistical significance for panels (**B**,**F**,**I**–**L**) was determined using an unpaired Student’s *t*-test. *p* < 0.01 (**).

**Figure 8:**
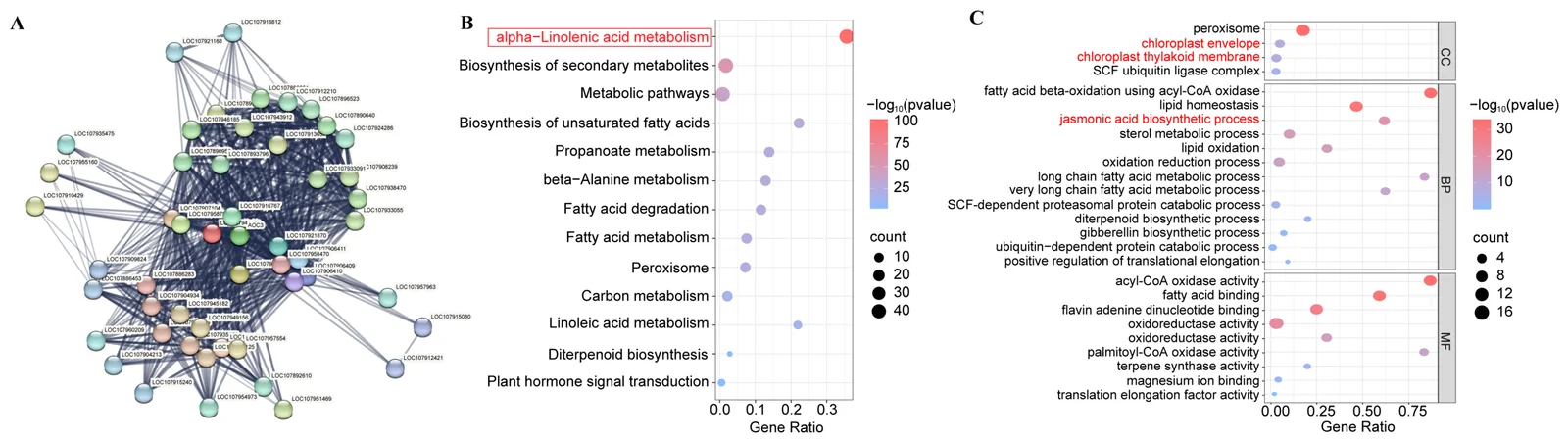
Predicted protein interaction network of GhAOC4/6. (**A**) Predicted proteins interacting with GhAOC4/6. Line thickness indicates interaction confidence. (**B**,**C**) KEGG (**B**) and GO (**C**) enrichment analyses of proteins predicted to interact with GhAOC4/6.

## Data Availability

The data supporting the findings of this study are available in the article and its Supplementary Information.

## Supplementary Materials

The following supporting information can be downloaded at: https://www.mdpi.com/article/10.3390/plants15192914/s1, Figure S1: Analysis of intraspecific collinearity analysis of the *AOC* genes in *Gossypium arboreum* and *Gossypium raimondii*; Figure S2: The physical locations of the *AOC* genes on the chromosomes in *Gossypium* species; Figure S3: Duplication patterns of *AOC* genes in *Gossypium* species; Figure S4: Conserved motif sequences of AOC in cotton; Figure S5: Three-dimensional structures of the AOC proteins from *Arabidopsis thaliana*, *Gossypium raimondii*, *Gossypium arboreum*, *Gossypium hirsutum* and *Gossypium barbadense*; Figure S6: Enrichment analysis of transcription factors potentially binding to the promoter regions of *Gossypium*; Figure S7: Multiple sequence alignment of the CDS of *GhAOCs*; Figure S8: Expression analysis of other *GhAOCs* and defense related genes in *GhAOC4/6* silenced cotton plants; Table S1: Characterization of *Gossypium hirsutum AOC* gene family; Table S2: All species information and download links; Table S3: Information on the members of the *AOC* family for 26 species; Table S4. *AOC* synteny gene pairs in ten dicots; Table S5: Duplication patterns of *AOC* genes in four *Gossypium* species; Table S6: Ka/Ks analysis of *AOC* gene pairs in four *Gossypium* species; Table S7: Prediction of *cis*-acting elements in the *AOC* genes promoter of cotton; Table S8: Prediction of transcription factor binding sites in the *AOC* promoters of *Gossypium*; Table S9: The FPKM value of expression of *AOC* genes in different tissues and under stresses in *Gossypium hirsutum*; Table S10: Sequence similarity analysis of *GhAOC4* and *GhAOC6* with other *GhAOC* family members; Table S11: Raw data of disease index and phytohormone content in the tested samples; Table S12: KEGG and GO enrichment analyses of putative GhAOC4/6 interacting proteins; Table S13: Primers used in the experiments; File S1: Multiple sequence alignment of AOC proteins from 26 selected species; File S2: Phylogenetic tree of AOC proteins from 26 selected species.
