# Supplementary material for: Evolutionary and Functional Characterization of the Allene Oxide Cyclase (AOC) Gene Family Reveals the Joint Contribution of GhAOC4/6 to Cotton Resistance Against Verticillium dahliae
Source: Plants (Basel). 2026 Sep 23;15(19):2914. doi: 10.3390/plants15192914 (PMC13666784; doi:10.3390/plants15192914)
Supplement: Supplementary file 1 [file plants-15-02914-s001.zip › plants-4530893-supplementary/Supplementary Figures.pdf]

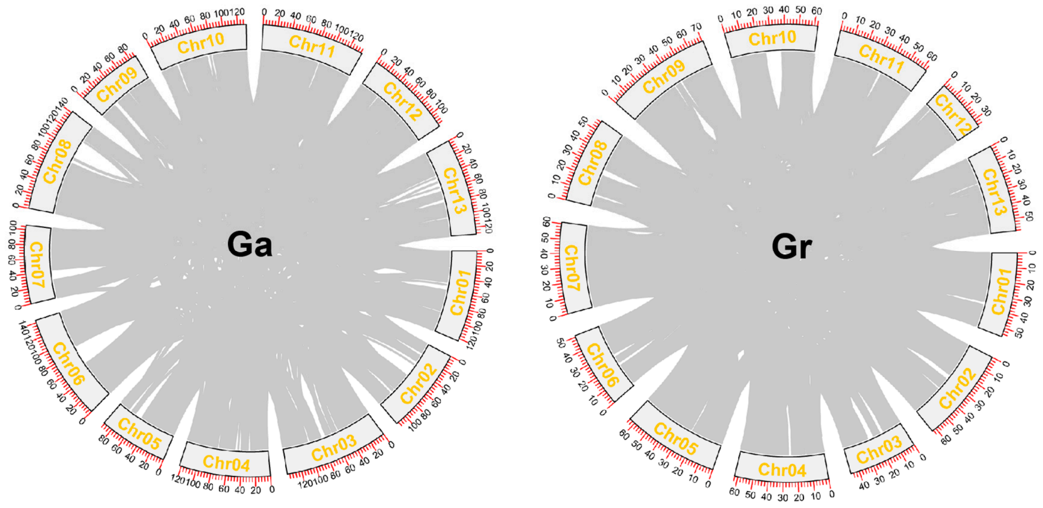


Figure S1. Analysis of intraspecific collinearity analysis of the *AOC* genes in *Gossypium* arboreum and *Gossypium raimondii*. The gray lines indicate regions of collinearity within species.

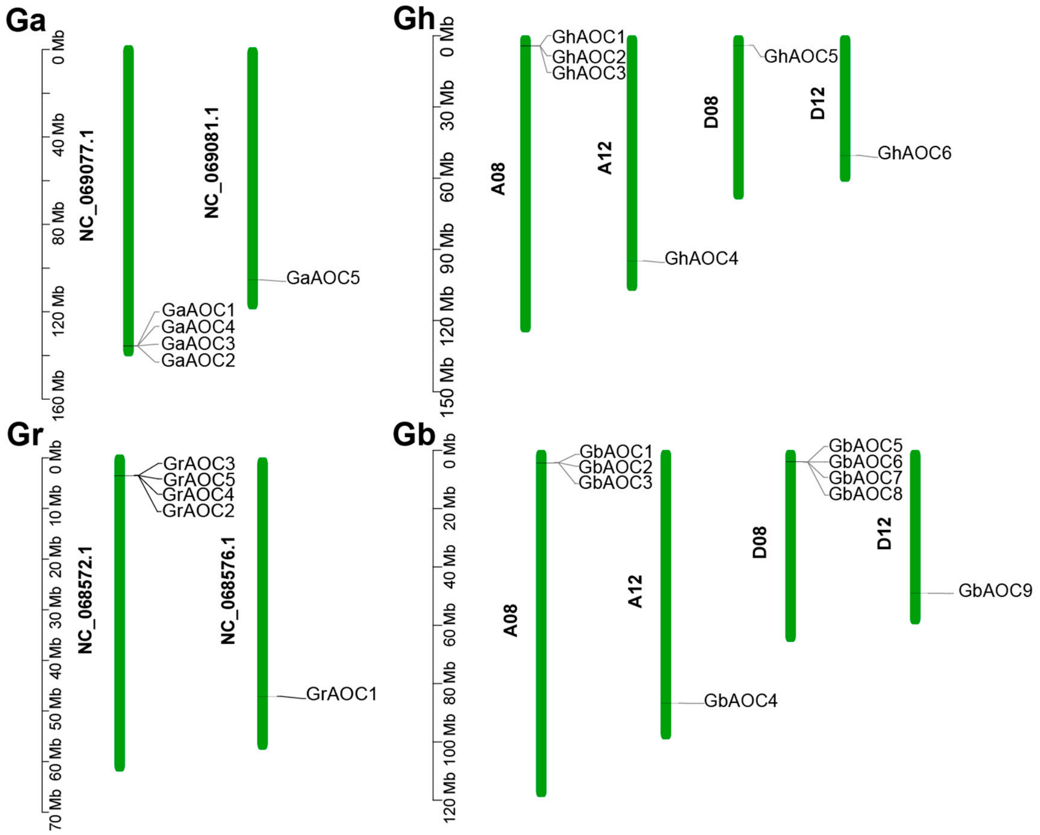


Figure S2. The physical locations of the *AOC* genes on the chromosomes in *Gossypium* species.

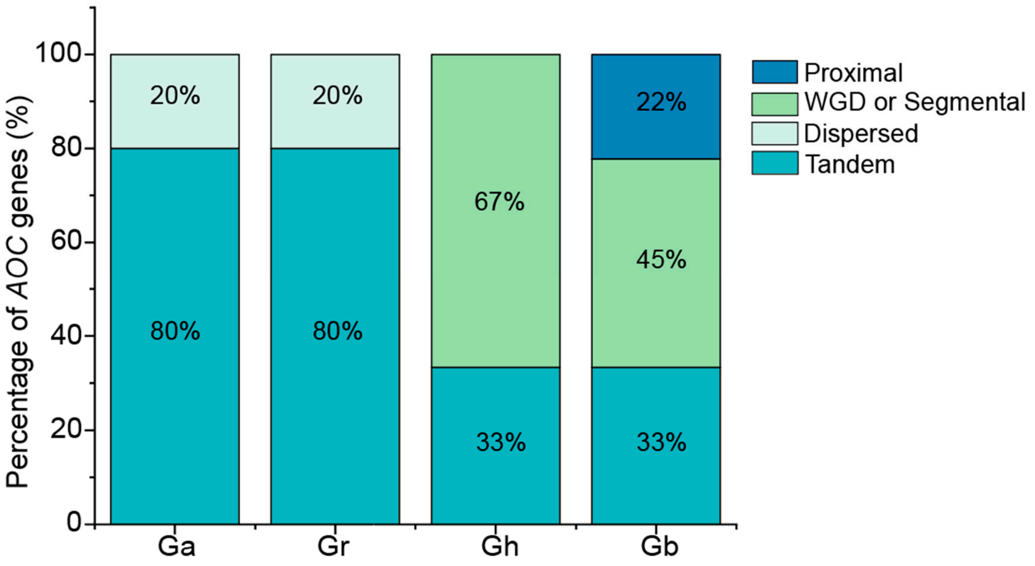


Figure S3. Duplication patterns of *AOC* genes in *Gossypium* species.

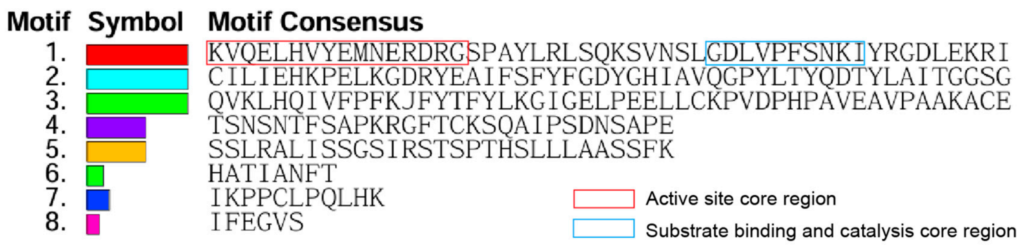


Figure S4. Conserved motif sequences of *AOC* in cotton.

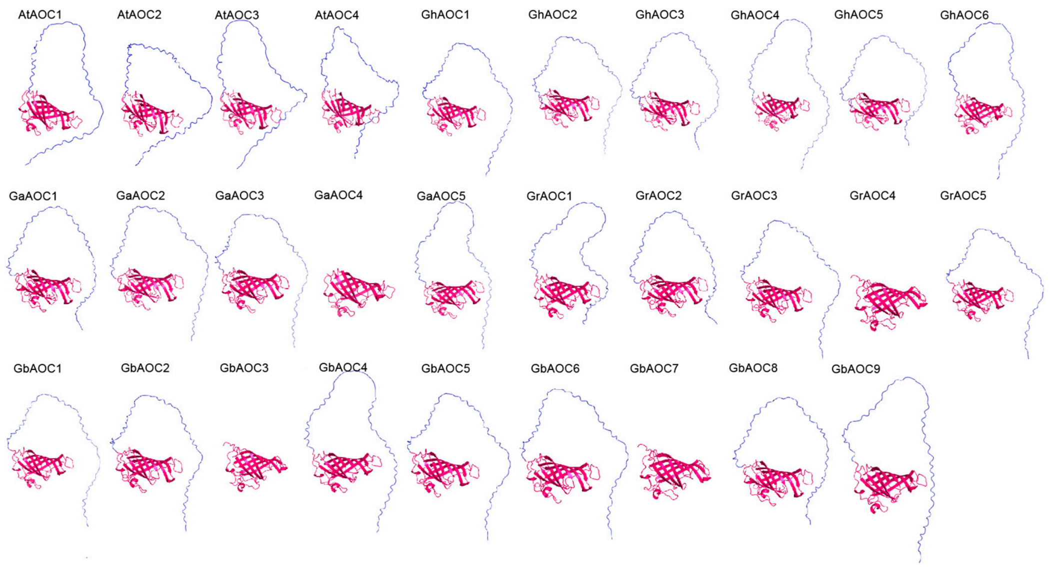


Figure S5. Three-dimensional structures of the AOC proteins from Arabidopsis thaliana,

*Gossypium raimondii*, *Gossypium arboreum*, *Gossypium hirsutum* and *Gossypium barbadense*.

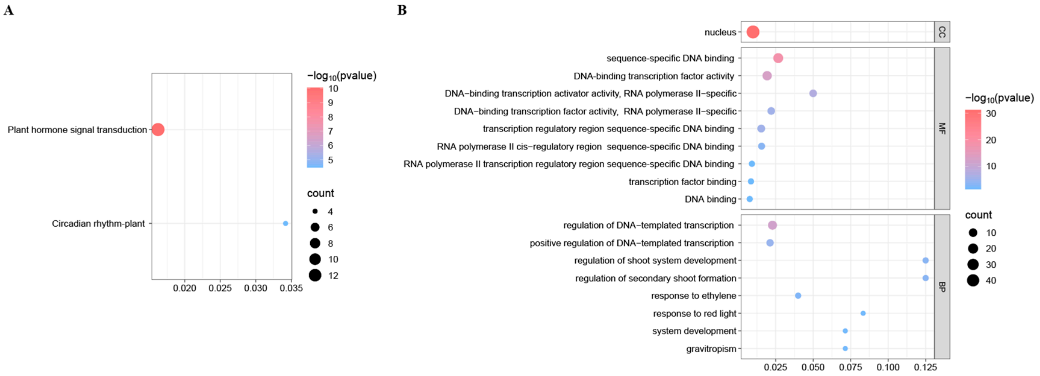


Figure S6. Enrichment analysis of transcription factors potentially binding to the promoter regions of *Gossypium*. (A-B) KEGG (A) and GO (B) enrichment analysis of transcription factors potentially binding to the promoter regions of *Gossypium*.

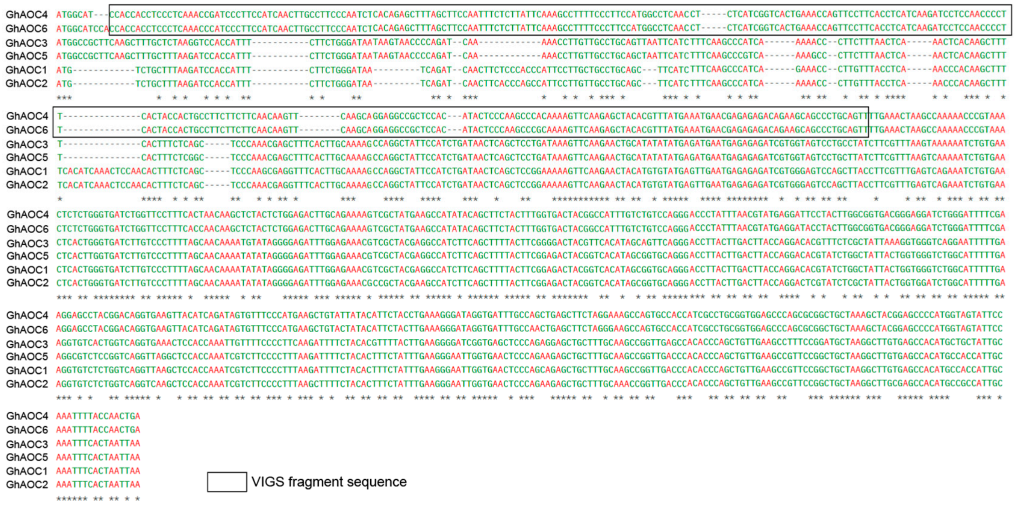


Figure S7. Multiple sequence alignment of the CDS of *GhAOCs*.The *GhAOC4/6* shared fragment used for VIGS is indicated by a black box.

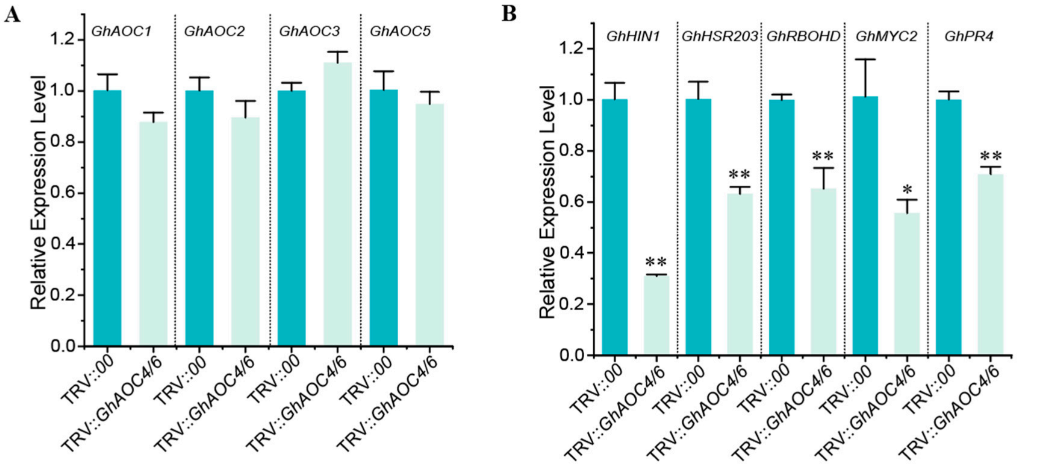


Figure S8. Expression analysis of other *GhAOCs* and defense related genes in *GhAOC4/6* silenced cotton plants. (A) Expression levels of other *GhAOC* genes in *GhAOC4/6* silenced and control plants. (B) Expression levels of the defense related genes *GhHIN1*, *GhHSR203*, *GhRBOHD*, *GhMYC2* and *GhPR4* in *GhAOC4/6* silenced and control plants before *V. dahliae* inoculation. *GhUBQ7* was used as the internal reference gene for normalization. Statistical significance was determined using an unpaired Student's *t*-test. $P < 0.05$ (*) and $P < 0.01$ (**).
